# Image Motion Extraction of Structures Using Computer Vision Techniques: A Comparative Study

**DOI:** 10.3390/s21186248

**Published:** 2021-09-17

**Authors:** Jau-Yu Chou, Chia-Ming Chang

**Affiliations:** Department of Civil Engineering, National Taiwan University, Taipei 10617, Taiwan; d06521008@ntu.edu.tw

**Keywords:** optical flow, digital image correlation, bilinear interpolation, phase-based motion magnification, Riesz pyramid, modal property extraction

## Abstract

Vibrational measurements play an important role for structural health monitoring, e.g., modal extraction and damage diagnosis. Moreover, conditions of civil structures can be mostly assessed by displacement responses. However, installing displacement transducers between the ground and floors in real-world buildings is unrealistic due to lack of reference points and structural scales and complexity. Alternatively, structural displacements can be acquired using computer vision-based motion extraction techniques. These extracted motions not only provide vibrational responses but are also useful for identifying the modal properties. In this study, three methods, including the optical flow with the Lucas–Kanade method, the digital image correlation (DIC) with bilinear interpolation, and the in-plane phase-based motion magnification using the Riesz pyramid, are introduced and experimentally verified using a four-story steel-frame building with a commercially available camera. First, the three displacement acquiring methods are introduced in detail. Next, the displacements are experimentally obtained from these methods and compared to those sensed from linear variable displacement transducers. Moreover, these displacement responses are converted into modal properties by system identification. As seen in the experimental results, the DIC method has the lowest average root mean squared error (RMSE) of 1.2371 mm among these three methods. Although the phase-based motion magnification method has a larger RMSE of 1.4132 mm due to variations in edge detection, this method is capable of providing full-field mode shapes over the building.

## 1. Introduction

Structural health monitoring (SHM) assists engineers to evaluate structural conditions through a scientific approach. In many SHM applications, vibrational measurements are recorded through sensors (e.g., accelerometers and displacement transducers) to understand the dynamic behavior of a structure [1,2]. In addition, displacement measurements, specifically the inter-story drifts, can be used to directly diagnose structural conditions [3]. Therefore, precise vibrational displacement measurements are needed in the condition assessment of buildings.

Although displacement measurements can be employed to directly evaluate the structural soundness, installing displacement transducers would result in great challenges such as finding an appropriate reference point [2]. Additionally, accelerometers are commonly installed on important components such as columns or beams to measure dynamic responses. However, sensor deployments are often costly and labor-intensive due to the high complexity and large size of typical civil structures [4]. Additionally, when testing laboratory-scale models, the self-weight of installed sensors may distort the measured responses, resulting in the misrepresentation of dynamic behavior [5]. Alternatively, vision-based techniques can be another good option to acquire vibrations of structural components [6].

With the aid of computer vision techniques, structural motions can be extracted through videos. When structures undergo in-plane motions, target movements will yield gradual intensity changes from pixel to pixel, where targets can be any recognizable components (e.g., beam-column connections) or manmade patterns adhered on structures (e.g., checkerboards). These intensity changes are viewed as optical flow and can be employed to extract motion velocities [7]. In the literature, Zhu et al. [8] proposed a marker-free method for displacement measurements using optical flow. The bolt at the main span of a scaled bridge was tracked by a smart phone with 4K resolution to measure displacement, with an average root mean square error (RMSE) of 0.2 mm. Won et al. [9] and Dong et al. [10] combined deep learning with optical flow methods to estimate the displacements from recorded videos of structures. In a study conducted by Won et al. [9], the performance using a 4K resolution camera was tested to have an RMSE of 0.07 mm for measurements less than 0.1 mm. In a study conducted by Dong et al. [10], the displacement of a model grandstand was measured using an industrial high-performance camera, resulting in a normalized RMSE of 0.87 mm. However, optical flow is sensitive to regions with significant and abrupt displacement variations and can only estimate motions of discrete regions [11].

Alternatively, digital image correlation (DIC) tracks the region of interest (ROI) through the correlations between two image portions, and the region that has the highest correlation with the ROI in the sequential frames can estimate the ROI motions [12]. In addition, DIC is more robust to brightness variations because the method computes the correlations between regions instead of estimating through pixel intensity. In applications, DICs are usually combined with speckle pattern coatings attached to the structural surface, and accurate full-field displacements are then obtained [13,14,15]. The vibrational measurements obtained from DIC can also be used for modal property extraction [16,17,18]. Moreover, interpolations within pixels can be employed in DIC methods to improve the measurements [19]. For instance, Luu et al. [20] applied DIC with the B-Spline interpolation, and the measurement accuracy to subpixel precision was upgraded. Zhao et al. [21] exploited DIC with a corrected three surface fitting algorithm (CTSFA) to improve the displacement estimation performance. Although DIC can be utilized to acquire full-field displacements from spackle pattern surfaces on structures, the method can also be applied to measure specific ROIs that have significant feature points.

Additionally, phase-based motion magnification is also one of the popular techniques to obtain vibrational displacements. Phase-based motion magnification manipulates the local phase differences within frames to enlarge the motion of a specific frequency band [22]. The magnified motion can enhance extracted pixel-precision movements to subpixel precision and does not require surface coatings. In the literature, Cha et al. [23] combined phase-based motion magnification with unscented Kalman filters to measure displacements of a laboratory-scale beam structure. The results showed that this approach had larger noise compared to laser vibrometers and accelerometers; however, the noise can be reduced by the unscented Kalman filter. Harmanci et al. [24] employed the phase-based motion magnification method to estimate displacements of a three-story frame structure. The resulting mode shapes were consistent with those identified from LVDTs. Moreover, full-field modal responses and modal properties can be extracted from the magnified motions [5,25,26]. In summary, the phase-based motion magnification method has the least requirements on the structural setting (i.e., without any coatings or targets) as well as can efficiently measure full-field displacements.

The objective of this study is to investigate the effectiveness of different motion extraction methods using a commercially available camera, including the optical flow with the Lucas–Kanade method, the digital image correlation (DIC) with bilinear interpolation, and the in-plane phase-based motion magnification using the Riesz pyramid. In Section 2, the methodology of the three approaches is introduced in detail. In Section 3.1, an experiment of a four-story steel-frame building structure using shake table testing is conducted to evaluate the displacement estimation performance using a commercially available camera. In Section 3.2, the performance and measurement errors of all the displacement extraction methods are discussed, and some improvements for each method, i.e., usage of reference frame, bilinear interpolation, and motion magnification, are employed and added to these methods. In Section 3.3, the estimated motions from three methods are then converted from pixels into millimeters using the relationship between the image and known parameters (i.e., story width). The estimated displacements are also compared to the measured displacements from linear variable displacement transducers (LVDT). In addition, the computational demand is compared. In Section 3.4, comparisons on modal properties are carried out by system identification to the obtained motions from the three methods. In Conclusions, the three methods are experimentally verified to have comparable displacement estimation performance with LVDTs, and the estimated displacements have sufficient precisions for modal property identification.

## 2. Computer Vision-Based Motion Extraction Techniques

In this study, three types of computer vision-based motion extraction techniques are briefly introduced, including using optical flow with the Lucas–Kanade method, the digital image correlation with bilinear interpolation, and the phase-based motion magnification using the Reisz pyramid.

### 2.1. Optical Flow with Lucas–Kanade Method

Optical flow is a common approach to detecting object motions besides counting the movement of certain pixels, which can only see motions that are larger than one pixel. In an image, objects are not constructed with sharp edges, instead, the images are blurred when transiting objects to objects. Additionally, movements represented in videos are converted as gradient colors. Thus, by combining the information of the pixel movement and the color change, the motion extracted from the video can be more accurate with a precision smaller than one pixel. As mentioned in Singh [7], a small motion of a pixel between two images can be represented as

(1) I(x,y,t)=I(x+Δx, y+Δy, t+Δt)

where  I is the intensity of a voxel at (x,y,t); x and y denote the positions in the coordinates of an image; t denotes the time step; and Δ represents the small difference in terms of frame or time. By using the local Taylor series approximation, Equation (1) is expanded as

(2) $$I\left(x+\Delta x,\;y+\Delta y,\;t+\Delta t\right)≅I\left(x,y,t\right)+\frac{\partial I}{\partial x}\Delta x+\frac{\partial I}{\partial y}\Delta y+\frac{\partial I}{\partial t}\Delta t$$

where $\;\frac{\partial I}{\partial }$ is the partial derivative of the intensity I. Note that the higher terms in the Taylor series are neglected. By truncating the higher order terms of the Taylor series approximation, the intensity increment satisfies

(3) $$\frac{\partial I}{\partial x}\Delta x+\frac{\partial I}{\partial y}\Delta y+\frac{\partial I}{\partial t}\Delta t=0$$

By dividing Equation (3) by Δt, the equation can be rewritten as

(4) $$I_{x}V_{x}+I_{y}V_{y}=-I_{t}$$

where $V_{x}$ and $V_{y}$ are the velocity in the *x* and *y* directions; and $I_{x}$, $I_{y}$, and $I_{t}$ are the derivatives of *I* with respect to *x*, *y*, and *t*. To solve the aperture problem in Equation (4), the Lucas–Kanade method [27] is applied to generate additional constraints. Given a target pixel, the image contents of two sequential frames near the target are assumed to be constant. With considerations of *n* pixels that are near the target, the local velocity vector satisfies

(5) $$J^{T}Jv=J^{T}b$$

where

$$J=\left[\begin{array}{cc} I_{x}\left(x_{1},y_{1}\right) & I_{y}\left(x_{1},y_{1}\right)\\ I_{x}\left(x_{2},y_{2}\right) & I_{y}\left(x_{2},y_{2}\right)\\ \vdots & \vdots \\ I_{x}\left(x_{n},y_{n}\right) & I_{y}\left(x_{n},y_{n}\right) \end{array}\right],\;v=\left[\begin{array}{c} V_{x}\\ V_{y} \end{array}\right],\;b=\left[\begin{array}{c} I_{t}\left(x_{1},y_{1}\right)\\ I_{t}\left(x_{2},y_{2}\right)\\ \vdots \\ I_{t}\left(x_{n},y_{n}\right) \end{array}\right]$$

where the velocity vector can be determined using the least-squares method. Finally, the displacements are integrated by

(6) $$d=\int_{0}^{t}vdt$$

where d is the displacement in both *x* and *y* directions.

### 2.2. Digital Image Correlation with Bilinear Interpolation

Digital image correlation (DIC) [28] is one of the popular motion tracking methods that can accurately measure the movements between sequential frames. The main concept of DIC is to compare the surface features (e.g., speckle patterns on structural surfaces) and to calculate the correlation of the feature regions within two frames. In small displacements, the target object can be assumed to be rigid, and the motion is, therefore, tracked by extracting the position that has the highest correlation as compared to the target. The extracted motion is restricted to the distance between the camera and target because the resolution would degrade as the camera moves further from the target.

**Figure 1:**
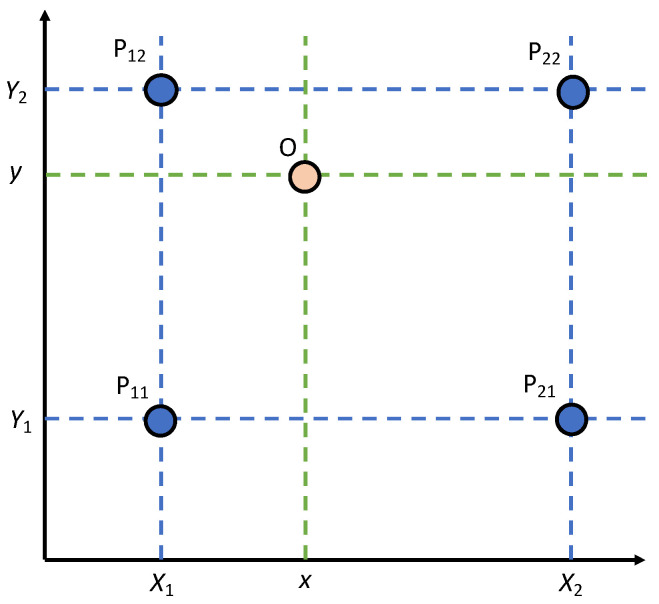
Illustration of the bilinear interpolation.

To improve the resolution of the video for long-distance filming, subpixel estimation, which can be accomplished using bilinear interpolation within four neighboring pixels, is applied to each frame as a pre-processing step before applying DIC [21]. As civil engineering applications usually focus on one-directional vibration response and neglects vertical responses. Figure 1 illustrates an example of the bilinear interpolation, where four pixels $P_{ij}\;\left(i\in \left[1,2\right],j\in \left[1,2\right]\right)$ are located at ($X_{1},Y_{1}$), ($X_{2},Y_{1}$), ($X_{1},Y_{2}$), and ($X_{2},Y_{2}$). As mentioned in Zhao et al. [21], the intensity of these four pixels is represented as $O\left(P_{ij}\right)$, and the intensity of intersection O(x) between $P_{1}$ to $P_{3}$ and $P_{2}$ to $P_{4}$ can be represented by

(7) $$O\left(x,y\right)=\frac{1}{\left(x_{2}-x_{1}\right)\left(y_{2}-y_{1}\right)}\left[x_{2}-x\;\;\;x-x_{1}\right]\left[\begin{array}{cc} O\left(P_{11}\right) & O\left(P_{12}\right)\\ O\left(P_{21}\right) & O\left(P_{22}\right) \end{array}\right]\left[\begin{array}{c} y_{2}-y\\ y-y_{1} \end{array}\right]$$

After obtaining the subpixel intensities, the correlation of a region of interest (ROI) of two frames is calculated by

(8) $$C={ℱ}^{-1}\left\{ℱ\left\{ROI_{1}\right\}\cdot \Delta ℱ{\left\{ROI_{2}\right\}}^{*}\right\}$$

where $ROI_{1}$ and $ROI_{2}$ are the intensities within the ROIs that correspond to the original frame and the deformed frame, respectively; ℱ{} is the frequency response using discrete Fourier transform; $ℱ{\left\{\right\}}^{*}$ is the Hermitian transpose of ℱ{}; and C is the correlation between $R_{1}$ and $R_{2}$. Thus, the integer shift (i.e., movement) can be obtained by seeking the maximum correlation such as

(9) $$\left(\Delta x,\;\Delta y\right)=\arg\underset{\left(x,y\right)}{\max}\left\{C\right\}$$

### 2.3. In-Plane Motion Magnification

The phase-based motion magnification method using the Riesz pyramid is capable of full-field, target-free measurement [29]. As mentioned in the paper, the input sub-band *I* and the Riesz transform from *I*, $R_{1}$, and $R_{2}$ are represented using the local phase φ, the local orientation θ, and the local amplitude *A* given by

(10) $$I=A\cos\left(\varphi \right),\;R_{1}=A\sin\left(\varphi \right)\cos\left(\theta \right),\;R_{1}=A\sin\left(\varphi \right)\sin\left(\theta \right)$$

The local phase φ can be presented in the complex domain by

(11) $$Ae^{i\varphi }=I+iQ$$

where Q is the quadrature pair when the Riesz transform is steered to the local dominant orientation. When a small motion is induced by a two-dimensional oriented sinusoid, the sub-band intensity can be written as

(12) $$I_{s}\left(x,y,t\right)=A\cos\left(\omega_{x}\left(x-\delta \left(t\right)+\omega_{y}y\right)\right)$$

where $I_{s}$ is the intensity of the sub-band image; *A* is the local amplitude; δ is the small horizontal motion; and $\omega_{x}$ and $\omega_{y}$ are the frequencies of the two-dimensional sinusoid. The quadrature pair can now be given by

(13) $$Q\left(x,y,t\right)=Asin\left(\omega_{x}x+\omega_{y}y-\omega_{x}\delta \left(t\right)\right))$$

From Equation (10), the local phase is written as

(14) $$\varphi =\omega_{x}x+\omega_{y}y-\omega_{x}\delta \left(t\right)$$

After applying temporal filters to remove the DC component $\omega_{x}x+\omega_{y}y$, the local phase can be magnified by β. With the local amplitude assumed to be constant, the motion-magnified sinusoid is given by

(15) $$I_{m}=A\cos(\omega_{x}\left(x-\left(1+\beta \right)\delta \left(t\right)+\omega_{y}y\right)$$

where $I_{m}$ is the sub-band intenstiy after magnified motion. More details can be found in a study conducted by Wadhwa et al. [29].

## 3. Experiment Verification

In this section, a scaled four-story steel-frame building is employed to experimentally evaluate the three motion extraction methods, which yield dynamic floor displacements from recorded videos. The measurements are converted from pixels to millimeters and compared to LVDT readings. In addition, the modal properties are identified from these displacement responses and also compared to the identification results from LVDTs. The performance and considerations of these three methods are eventually discussed.

### 3.1. Experimental Setup

**Figure 2:**
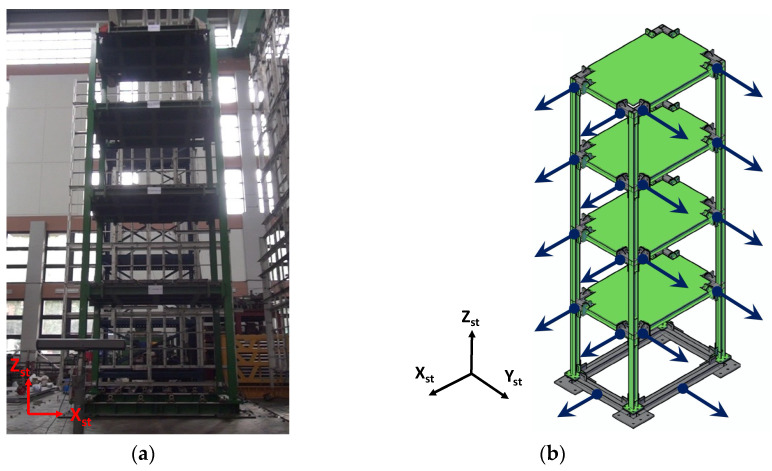
Experimental setup: (**a**) photo captured by camera; (**b**) LVDT locations.

In this study, a scaled four-story steel-frame building is excited by the shake table at the National Center for Research on Earthquake Engineering (NCREE) in Taiwan, and the effectiveness of the three motion extraction methods are evaluated. The methods include the optical flow, the digital image correlation with subpixel estimation, and the phase-based motion magnification using Riesz Pyramid. Figure 2 represents the specimen and sensor locations used in the experiment. In this building, the H-beams used have a cross section of 150 mm×150 mm, and these H-beams are utilized as the structural columns and beams. The story height, width, and weight are 2.2 m, 3.15 m, and 6 tons, respectively.

In the experimental setup, four LVDTs per floor are installed to measure absolute displacements in both the *x* and *y* directions, and a total of 16 LVDTs are installed on this building as shown in Figure 2b. Two additional LVDTs are located at the base to measure ground displacements under the 50-gal white noise excitation. Note that this building is excited along the *x*_st_ direction during the experiment, but imperfect fabrication would induce very small floor displacements in the *y*_st_ direction. Thus, this study only presents LVDT readings in the *x*_st_-direction. The duration is roughly 90 s. In addition, a commercially available camera is placed on a fixed floor, i.e., the outside of the shaking table area, to record motions of the building and shake table. The distance between the structure and the camera is around 3 m, with a 30-fps framerate and a resolution of 1080×1920 pixels. Both the building and camera parameters are summarized in Table 1.

### 3.2. Motion Extraction

#### 3.2.1. Optical Flow with the Lucas–Kanade Method

**Figure 3:**
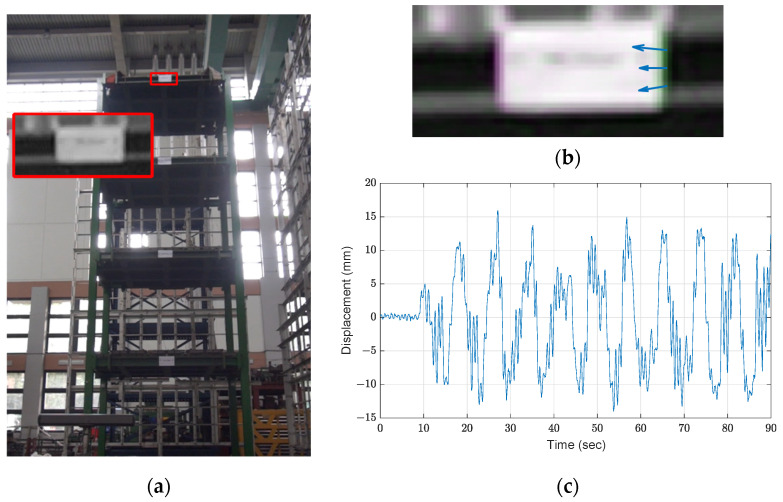
Results of optical flow with the Lucas–Kanade method: (**a**) ROI; (**b**) estimated optical flow; (**c**) roof displacement.

Figure 3a demonstrates the selected ROI for the roof floor measurement. Although the selected portion seems to have a low resolution, sufficient features (e.g., the edges of the white paper) are obtained for motion extraction using optical flow with the Lucas–Kanade method. As shown in Figure 3b, the estimated velocity is acquired from the feature points. As the structure does not move in the *z*_st_-direction, only horizontal velocities are utilized to retrieve the motion. The displacement time history is calculated by integrating the velocity over time and converted to physical units by using the relationship between the story width in pixels and the exact width. This relationship is 7.8 mm/pixel, and the result is exhibited in Figure 3c.

**Figure 4:**
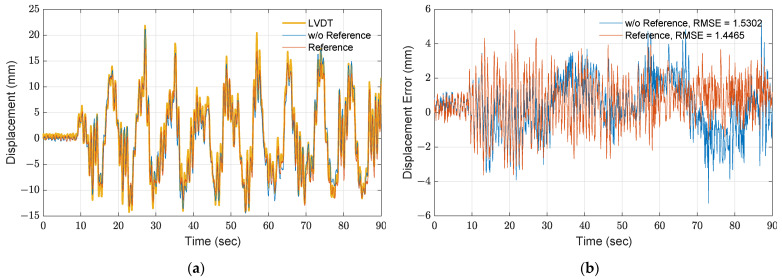
Comparison of different optical flow estimation approaches: (**a**) time history; (**b**) errors.

Two motion tracking approaches by optical flow are established and examined such as (1) by estimating the optical flow from sequential frames (i.e., the current and previous frame) and (2) by comparing each frame with the reference frame (i.e., the first frame). As found in the experimental results, one disadvantage of estimating the optical flow by matching the previous frame is that the errors may be enlarged and accumulated if errors exist in the previous steps [9]. Figure 4 shows the comparison between the LVDT response and the two approaches, where the yellow line indicates the measurement of the roof from the LVDT and the blue and orange curves indicate the displacement obtained from the first and second approaches. Note that the LVDT measurements are down sampled to 30 Hz for comparison with these two approaches. To understand the accuracy of these approaches, the root mean square error (RMSE) is computed by

(16) RMSE=∑j=1N(djLVDT−djestimated)2N

where $d_{j}^{\mathrm{LVDT}}$ and $d_{j}^{\mathrm{estimated}}$ are the measurement from the LVDT and optical flow approaches at the *j*-th time step and *N* is the total number of time steps. As shown in Figure 4b, the optical flow using a reference frame has a smaller RMSE of 1.44 mm. Although the two approaches are capable of estimating displacement with high accuracy, the estimation using the optical flow with a reference frame shows a lower RMSE.

#### 3.2.2. Digital Image Correlation with Bilinear Interpolation

**Figure 5:**
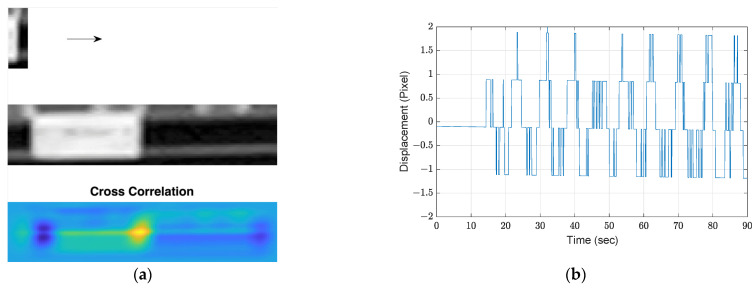
Motion extraction from DIC: (**a**) procedure of cross correlation; (**b**) extracted displacement.

Digital image correlation extracts motion by seeking the pixel location of the image portion where the maximum correlation with the ROI is achieved. The motion can then be determined by calculating differentiated locations from the first frame in the pixel coordinates. However, small motions are difficult to be distinguished and to extract good-quality displacements. By the same ROI used in Section 3.2.1, Figure 5a illustrates the calculated correlations by comparing a feature portion with the ROI. The light-yellow region presents the highest correlation. Then, using this DIC approach, the resulting displacement is shown in Figure 5b. As seen, the displacement in pixels only provides the motion trend without useful information that can be further analyzed.

**Figure 6:**
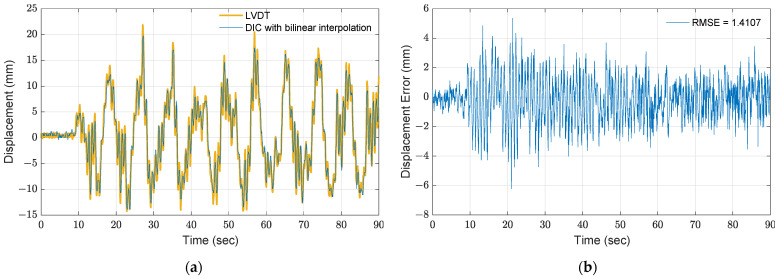
Results of DIC with bilinear interpolation: (**a**) comparison between the proposed method by a 20-subpixel interpolation and LVDT measurement; (**b**) error.

Alternatively, using bilinear interpolation within the ROI generates subpixels that can increase precision for the DIC method. In the bilinear interpolation, one pixel is interpolated into 20 subpixels and then employed to track the roof displacement. Next, the displacement in pixels is converted to physical units by using the story width in pixels and the exact length in millimeters. To reduce the computational load, only a small portion of the ROI is utilized for bilinear interpolation, and the motions in the *z*_st_-direction are assumed to be 0. As shown in Figure 6a, the extracted displacement in the physical unit matches the LVDT measurement, where the RMSE is 1.41 mm. Moreover, the accuracy is improved by 44.7%, and precision is improved from 7.8 to 0.39 mm/pixel, as compared to Figure 5b.

#### 3.2.3. Phase-Based Motion Magnification Using the Riesz Pyramid

**Figure 7:**
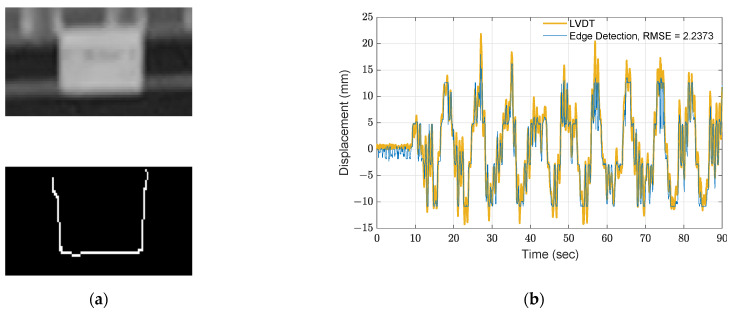
Motion extraction using edge detection: (**a**) Canny’s edge detector; (**b**) estimated displacement at roof.

Another approach is to track motion with edge detection. By averaging the pixel changes in each row, subpixel precision of the measurements can be reached. However, the measured displacement still contains a large RMSE and may not be useful for dynamic feature extraction. As shown in Figure 7, the edge determined using the Canny’s edge detector [30] with a recommended threshold of 0.8 is utilized, and the extracted motion is converted to millimeters. In Figure 7b, the extracted roof motion is also compared to the displacement measurement using LVDT, and the resulting RMSE is 2.24 mm, which is much higher than the results obtained from optical flow with the Lucas–Kanade method and DIC with bilinear interpolation.

**Figure 8:**
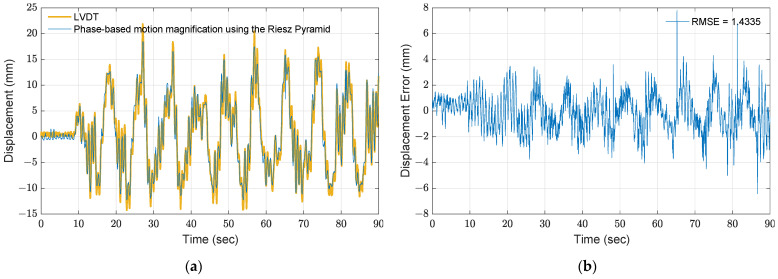
Results of phase-based motion magnification using the Riesz pyramid: (**a**) comparison between the proposed method with magnification factor of 5 and measurement from LVDT; (**b**) error.

To improve the accuracy, the local phases of the pixels are determined using the Riesz pyramid, and the magnified motions are manipulated. One advantage of using the Riesz pyramid is to magnify motions without dividing a single pixel into multiple subpixels. In the pyramid reconstruction process, a frequency band of 0 to 15 Hz is selected in accordance with the Nyquist frequency. The magnification factor β in Equation (15) is set to five. Note that a larger magnification factor can enlarge motions to be more visible; however, the magnified motions would be blurry. The resulting magnified responses are then utilized to extract displacement using the Canny’s edge detector. Figure 8 shows the results using the proposed method. As seen in Figure 8b, the RMSE is 1.433, which is lower than the result by the direct use of the edge detection. Much better performance is achieved by combining the phase-based motion magnification with the edge detection.

### 3.3. Discussions of Motion Extraction Results

In this section, three proposed motion extraction methods, including the optical flow with the Lucas–Kanade method, the DIC with bilinear interpolation, and the phase-based motion magnification using the Riesz pyramid, are compared. In the optical flow with the Lucas–Kanade method and DIC with bilinear interpolation, the reference adopted is the first frame in accordance with the findings in Section 3.2.1. Table 2 lists the RMSEs calculated using Equation (16), and the maximum displacement error (Error_max_) of all the floors. Additionally, the RMS_ref_ indicates the root mean squares of all the floor LVDT readings, and the maximum floor displacements (i.e., Disp_max_) are provided for the reference. In the evaluation of this section, the three approaches employed the same ROIs to investigate performance. Note that changing the ROIs can highly affect the results because the quality of the image portion may be affected by multiple reasons such as the light source, sufficient feature points, etc.

As found in the results, all three approaches have a similar RMSE of around 1.3 mm (20% of the RMS_ref_). Moreover, the RMSE on the fourth floor is intended to be larger than other stories for the three methods because the target is much further away from the camera. A distant target yields fewer or insufficient feature points in ROI to track motions. In addition, the RMSE of the first floor using the optical flow with the Lucas–Kanade method and the DIC with bilinear interpolation is slightly larger than using the phase-based motion magnification using the Riesz pyramid. As the phase-based motion magnification enlarges the motion instead of seeking for subpixel intensities, higher accuracy is achievable when processing small motions. Still, the performance of the phase-based motion magnification using the Riesz pyramid is affected by the edge detection results. For example, the accuracy will drop when the edges are not well detected, including the RMSE and the maximum displacement error.

In addition, the computational demand of the three approaches is compared per single-story motion estimation. The computer is configured with an Intel^®^ Core™ i7-8700 CPU and 48 gigabits of RAM. As listed in Table 3, the phase-based motion magnification using the Riesz pyramid has the minimum computational usage, and the DIC with bilinear interpolation is tested to be the most inefficient. This is because the bilinear interpolation needs to be calculated multiple times to generate subpixel intensities, and a cross-correlation matrix needs to be generated in each frame. Moreover, if more subpixels are produced from the ROI by bilinear interpolation, the computational load will rapidly increase.

### 3.4. System Identification

**Figure 9:**
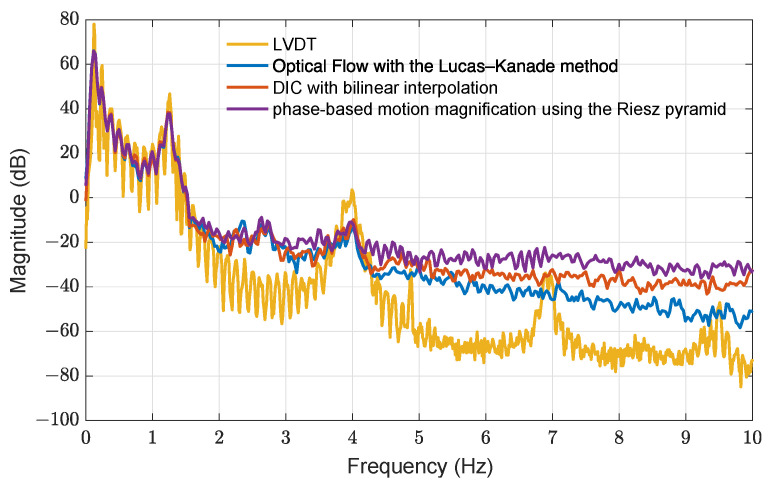
Power spectral density of roof displacement using the three motion extraction approaches and LVDT measurement.

Modal properties can be extracted from the proposed three approaches. Due to the limited sampling rate and the less accurate displacements, the number of identifiable modes is restricted. Figure 9 indicates the power spectral density of the roof displacement calculated by the three approaches and the LVDTs. As seen, only two modes can be observed from the computer vision-based methods, and the first four natural frequencies are 1.26, 3.99, 6.90, and 9.51 Hz using the peak picking method from the LVDT power spectral density.

**Figure 10:**
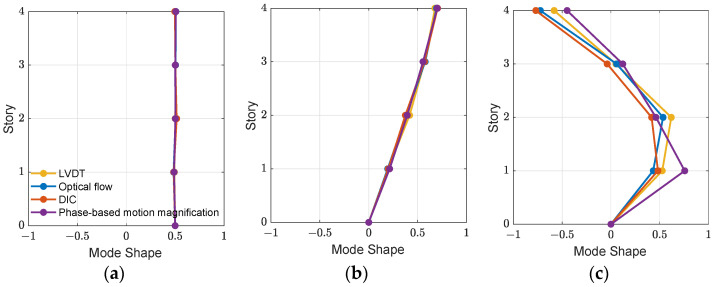
Identified mode shapes: (**a**) rigid-body mode; (**b**) 1st mode; (**c**) 2nd mode.

Figure 10 demonstrates the identified mode shapes of the first two modes and the rigid body motions in 0.1 Hz from four sorts of displacements. The mode shapes are identified using the frequency domain decomposition [31]. As seen in Figure 9, a local peak is found in 0.1 Hz from the LVDT power spectral density. After extracting the mode shape in this frequency, a rigid-body mode is identified. As the displacements estimated using the three computer vision-based approaches or measured by the LVDTs are presented in the fixed (or absolute) coordinates, i.e., the motions related to a fixed ground, this rigid-body mode exists and is identified from all sorts of displacements. Note that a rigid-body mode should be located in 0 Hz. Due to some unknown dynamics (e.g., connections between the building base and shake table), the resulting frequency is shifted to around 0.1 Hz.

As shown in Figure 10, the first mode shapes obtained using the three proposed approaches meet a good agreement with the identified result from the LVDTs. Although larger variations are observed in the second mode shapes of the three approaches, which is mostly due to the relatively high noise levels, the results are still comparable with the mode shape obtained from the LVDTs. Moreover, the comparison of the modal properties identified from four sorts of displacements are listed in Table 4. The results indicate that the computer vision-based methods are capable of identifying the natural frequency with a certain level of accuracy with an error of less than 2%. In addition, the mode shape accuracy is calculated through modal assurance criterion (MAC) [32] as

(17) $$\mathrm{MAC}=\frac{\varphi_{R}{}^{T}\varphi_{\mathrm{ID}}}{\left\|\varphi_{R}\right\|\left\|\varphi_{\mathrm{ID}}\right\|}$$

where $\varphi_{\mathrm{ID}}$ is the identified mode shape from the computer vision-based methods, and $\varphi_{R}$ is the mode shape by the LVDTs. The MACs of the two modes using three approaches are all over 0.9, indicating that the proposed approaches can yield estimated displacements that are identifiable for mode shapes with high accuracy.

**Figure 11:**
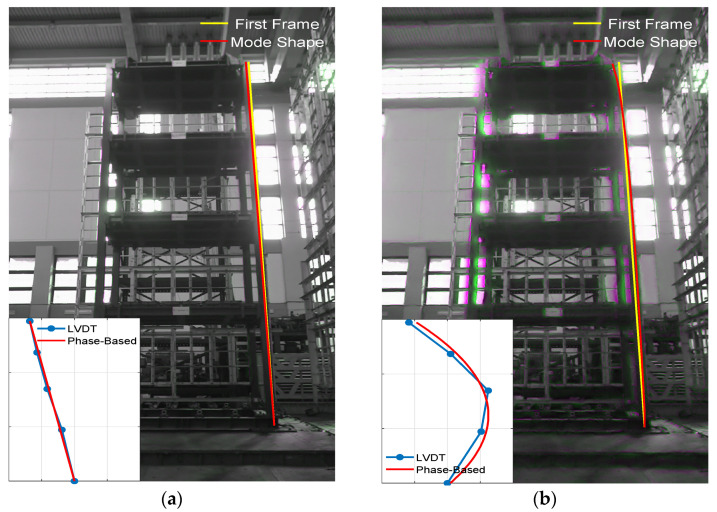
Identified full-field mode shapes from the phase-based motion magnification using the Riesz pyramid: (**a**) 1st mode; (**b**) 2nd mode.

In addition, because the phase-based motion magnification method is capable of enlarging a specific frequency band, the full-field modal responses are extracted [5]. The frequency band is determined by choosing appropriate upper and lower limits corresponding to the natural frequency. In this experiment, the frequency band of the first and second modes are between 1.11 and 1.35 Hz and between 3.59 and 4.39 Hz, respectively. The magnification factors are set to 50 and 500 for the first and second modes. Figure 11 shows the mode shapes extracted from the modal response. As seen here, the first two full-field mode shapes can be revealed and are quite comparable with the results from the LVDTs.

## 4. Conclusions

In this study, three computer vision-based motion extraction methods were investigated and experimentally verified by a four-story steel-frame building using shake table testing. These three approaches included the optical flow with the Lucas–Kanade method, the digital image correlation with bilinear interpolation, and the phase-based motion magnification using the Reisz pyramid, which were evaluated using a commercially available camera by the displacement acquiring accuracy and the extraction capability of modal properties. In the optical flow approach, the results indicated that estimating the flow using a reference frame can slightly improve the accuracy by about 5%. As for the digital image correlation (DIC) methods, the precision reached sub-pixel if bilinear interpolation was applied to the region of interest (ROI). For an interpolation factor of 20, the precision can be increased by 20 times, resulting in more detailed displacements to be obtained. The phase-based motion magnification method can estimate more accurate motions than the direct use of edge detection with an RMSE improvement of 38.7%. For the single-ROI motion extraction, the phase-based motion magnification method showed the largest average RMSE among the three approaches due to the error induced by edge detection. Still, the magnified motion effect in this method overcame the small motion problem (e.g., movements within a couple of pixels) in the optical flow and DIC methods. For example, the experimental results demonstrated the smallest RMSE in the estimated first floor displacement using this phase-based motion magnification method.

In the system identification results, the first two modes out of four modes were successfully identified by the displacements generated from these three computer vision-based methods. The maximum error of 2% was found in the natural frequency identification by the displacements from the phase-based motion magnification method, while the identified mode shapes all had MACs above 0.9 from the estimated displacements using these three methods. Additionally, the phase-based motion magnification method was able to extract full-field mode shapes with target-free measurements. As a result, the three methods were found to have a similar performance as compared to the LVDT measurements.

To sum up, the DIC method had the lowest average RMSE of 1.2371 mm, while the phase-based motion magnification method yielded the largest RMSE of 1.4132 mm. However, the interpolation process in the DIC method required additional computational demand as compared to the optical flow method, and the accuracy between these two methods were quite comparable, as seen in the experimental results. Moreover, by using a commercially available camera with a certain distance (i.e., 3 m away from the building) and image resolution (i.e., 1080 × 1920 pixels), the measured story displacement errors were all lower than 2 mm. In addition, the estimated displacements can be exploited to identify modal properties. As found in the experimental verification, all of these three methods produced modal properties of this building consistent with those identified from LVDTs.

## Figures and Tables

**Table 1:** Parameters for experimental setup.

Four-Story Steel-Frame Building		Camera System	
Story height	2.2 m	Resolution	1080×1920
Story width	3.15 m	Frame Rate	30 fps
Story weight	6 tons	distance	~3 m
Beam cross section	H-type 150 mm×150 mm		
Sensors	18 LVDTs		

**Table 2:** RMSE of each story measurement using the proposed three approaches.

	LVDT		Optical Flow		DIC		Phase-Based	
	Disp_max_	RMS_ref_	Error_max_	RMSE	Error_max_	RMSE	Error_max_	RMSE
1st Floor	16.96	6.5202	5.17	1.2661 (19.4%)	4.52	1.2193 (18.7%)	5.44	1.1034 (16.9%)
2nd Floor	19.35	6.8226	4.75	1.3124 (19.2%)	4.85	1.0652 (15.6%)	4.66	1.5071 (22.1%)
3rd Floor	20.24	7.0277	5.36	1.1202 (15.9%)	5.51	1.2534 (17.8%)	9.72	1.6095 (22.9%)
4th Floor	21.45	7.1746	5.02	1.4465 (20.2%)	7.16	1.4107 (19.7%)	5.15	1.4330 (20.0%)

Unit: mm.

**Table 3:** Computational load of a single-story motion extraction using the three approaches.

	Optical Flow	DIC	Phase-Based
Speed	45.94 s	56.30 s	11.86 s

**Table 4:** Comparison of modal properties using the proposed three approaches.

	Optical Flow		DIC		Phase-Based	
	$\omega_{n}$	MAC	$\omega_{n}$	MAC	$\omega_{n}$	MAC
1st Mode	1.23 Hz (−1.6%)	1.00	1.23 Hz (−1.6%)	1.00	1.23 Hz (−1.6%)	1.00
2nd Mode	4.01 Hz (+0.5%)	0.97	4.01 Hz (+0.5%)	0.92	3.92 Hz (−1.8%)	0.95
